# Soluble transferrin receptor can predict all-cause mortality regardless of anaemia and iron storage status

**DOI:** 10.1038/s41598-022-15674-w

**Published:** 2022-07-13

**Authors:** Minjung Kang, Soie Kwon, Whanhee Lee, Yaerim Kim, Eunjin Bae, Jeonghwan Lee, Jae Yoon Park, Yong Chul Kim, Eun Young Kim, Dong Ki Kim, Chun Soo Lim, Yon Su Kim, Jung Pyo Lee

**Affiliations:** 1 Department of Internal Medicine, Seoul National University Hospital, Seoul, Korea; 2 Department of Internal Medicine, Seoul National University College of Medicine, Seoul, Korea; 3 School of the Environment, Yale University, New Haven, CT USA; 4 Department of Internal Medicine, Keimyung University School of Medicine, Daegu, Korea; 5 Department of Internal Medicine, Gyeongsang National University Changwon Hospital, Changwon, Korea; 6 Department of Internal Medicine, College of Medicine, Gyeongsang National University, Jinju, Korea; 7 Department of Internal Medicine, Seoul National University Boramae Medical Center, Seoul, Korea; 8 Department of Internal Medicine, Dongguk University Ilsan Hospital, Goyang, Korea; 9 Department of Internal Medicine, Dongguk University College of Medicine, Gyeongju, Korea; 10 Research Center for Chronic Disease and Environmental Medicine, Dongguk University College of Medicine, Gyeongju, Korea; 11 Mental Health Center, Seoul National University Health Care Center, Seoul, Korea; 12 Departement of Human Systems Medicine, Seoul National University College of Medicine, Seoul, Korea

**Keywords:** Biomarkers, Medical research, Nephrology

## Abstract

Despite interest in the clinical implications of soluble transferrin receptor (sTfR), previous studies on the association of sTfR with mortality in the general population are lacking. Therefore, we analysed the association between sTfR and all-cause mortality in the general United States adult population. We conducted a prospective cohort study using National Health and Nutrition Examination Survey data from 2003 to 2010. A total of 5403 premenopausal nonpregnant females were analysed in this study. The mean age was 34.2 years (range 20.0–49.9 years). Participants were divided into log(sTfR) tertiles. The primary outcome was all-cause mortality. The secondary outcome was chronic kidney disease (CKD) development (composite of estimated glomerular filtration rate < 60 ml/min/1.73 m^2^ and/or random urine albumin-to-creatinine ratio ≥ 30 mg/g). During a median 8.7 years of follow-up, 103 (1.9%) participants died. Compared with the reference group (log(sTfR) 0.45–0.57), the highest tertile of log(sTfR) was associated with all-cause mortality (log(sTfR) > 0.57, hazard ratio [HR] 1.77 [95% CI 1.05–2.98]) in a multivariable hazards model including covariates such as haemoglobin and ferritin. Patients in the highest tertile of log(sTfR) also had an increased risk of CKD relative to those in the reference tertile. High sTfR was associated with all-cause mortality and CKD regardless of anaemia and iron storage status.

## Introduction

Transferrin receptor (TfR) is a homodimer linked to two identical transmembrane subunits and is expressed in all cell membranes except mature erythrocytes and some terminally differentiated cells^1^. Soluble transferrin receptor (sTfR) represents the extracellular domain part of the TfR^2^, and its concentration is proportional to the amount of TfR in the total body^1^. TfR is known to mediate the process by which iron in plasma binds to transferrin and is taken up to cells^3^, and the concentration of sTfR is increased in individuals with iron deficiency anaemia^4^. Therefore, sTfR is known as a marker of iron deficiency anaemia (IDA)^4^. Serum ferritin, as a marker of iron stores, could increase in the context of inflammation or chronic disorders, confusing the assessment of IDA^5,6^. In these cases, sTfR is helpful for assessing IDA. Another known role of sTfR is that it represents the erythropoietic activity of bone marrow^7^. sTfR increases in diseases associated with an increase in erythroid proliferation, such as autoimmune haemolytic anaemia and thalassemia, regardless of iron status. In addition, sTfR increases when erythroid activity increases due to increased oxygen demand in nonanaemic patients^8^. Collectively, sTfR could be a marker of erythroid activity regardless of iron status or anaemia.

A few studies have demonstrated a significant association between sTfR and chronic disease^9,10^. One longitudinal study showed that higher sTfR was associated with the risk of diabetes mellitus (DM) among overweight and obese participants^9^. In another cross-sectional study, it was shown that sTfR and the prevalence of chronic kidney disease (CKD) were correlated^10^. Moreover, studies on sTfR and mortality were mainly conducted in high-risk patients with cardiovascular disease^11–15^. However, analyses of sTfR and mortality in the general population are rare. One study of 1,864 individuals from the general German population showed that high sTfR was associated with all-cause mortality and cardiovascular mortality^16^. However, this study was restricted to specific races and hospital-based participants scheduled for coronary angiography.

We conducted a prospective cohort study in the general United States (US) adult population with variable races using data from the National Health and Nutrition Examination Survey (NHANES) from 2003 to 2010. We sought to investigate the association between sTfR and all-cause mortality.

## Materials and methods

### Study participants

We analysed 5,403 adult females (aged 20 − 49 years) who participated in NHANES from 2003 to 2010. Because sTfR was measured in nonpregnant women (aged 15 − 49 years) in the NHANES dataset and adolescents tend to have a high sTfR^17^, we excluded participants aged < 20 years (*n* = 2152). Participants were also excluded if they did not have sTfR data (*n* = 570) or had extreme sTfR values (> 20 mg/L). Ultimately, 5,403 females were included, excluding subjects who were lost to follow-up (*n* = 9) (Supplemental Fig. 1). This cohort study complied with the Declaration of Helsinki. Because the NHANES dataset was available for public download, this study was exempted from the review by the institutional review board of Seoul National University College of Medicine (E-2106–050-1225). All methods were carried out in accordance with relevant guidelines and regulations.

**Figure 1:**
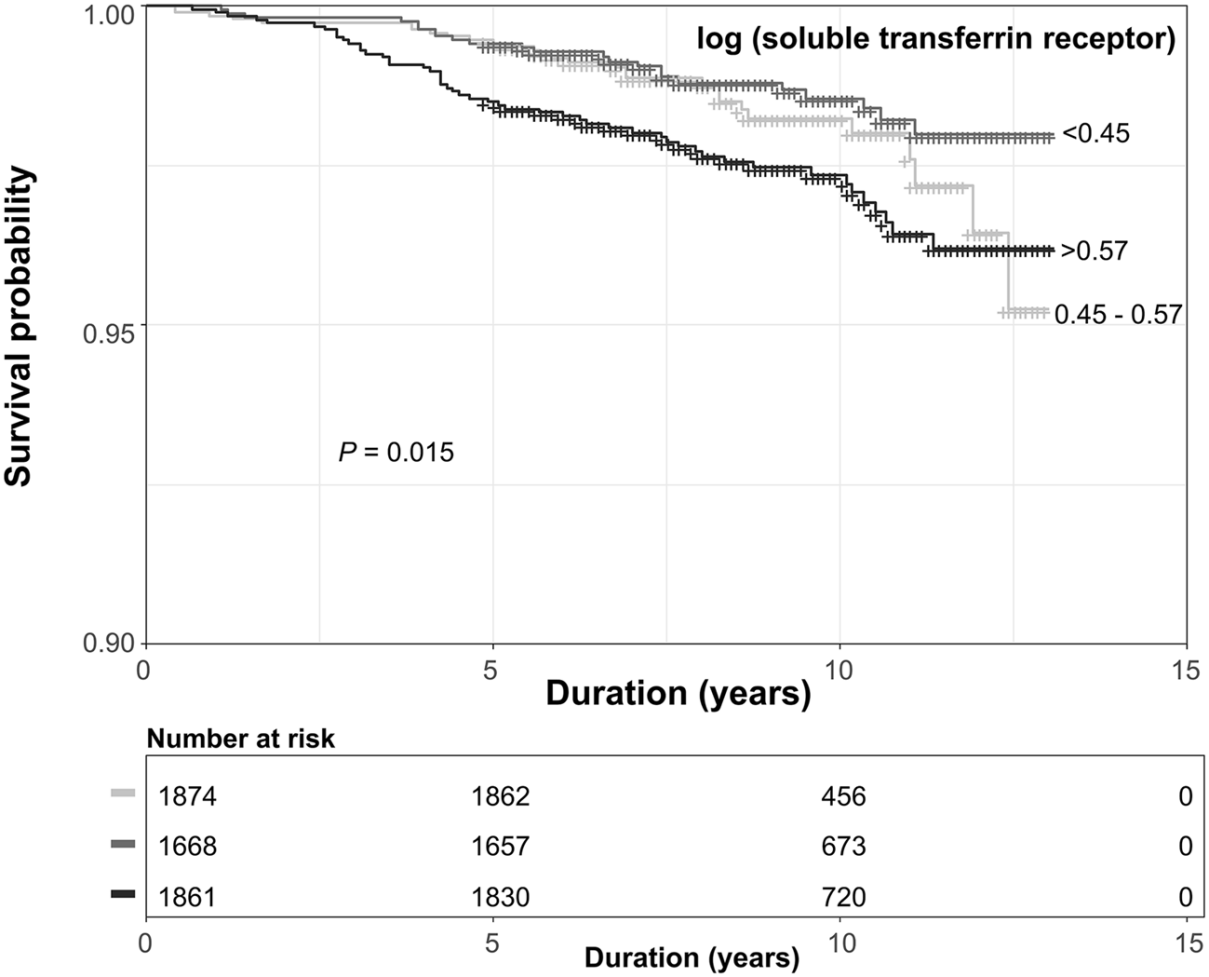
Cumulative survival curve according to tertile of log(soluble transferrin receptor). Kaplan–Meier curves showed that the highest tertile of log(soluble transferrin receptor) had the lowest survival probability for all-cause mortality.

### Data collection and definitions

Information regarding participant demographic information, medical history, laboratory values, and mortality was obtained from the NHANES database^18^. sTfR was measured by immunoturbidimetry using Roche kits (Roche Diagnostics, Mannheim, Germany)^19^ on a Hitachi 912 clinical analyser (NHANES 2003–2008) and on a Hitachi Mod P (NHANES 2009–2010). The results obtained from the 2 instruments have been shown to be comparable^20^. Serum ferritin was measured by a BioRad assay (NHANES 2003), by a Roche/Hitachi assay (NHANES 2004–2008), and by a Roche Elecsys assay (NHANES 2009–2010). Serum and urine creatinine levels were measured using the Jaffe rate method. These analyses were calibrated by an isotope dilution mass spectrometry reference method. Urine albumin was measured by solid-phase fluorescent immunoassay. The estimated glomerular filtration rate (eGFR) was calculated by the CKD Epidemiology Collaboration equation ^21^. Participants were divided according to tertile of log(sTfR).

DM was defined as one or more of the following: (1) a history of DM; (2) fasting glucose level > 126 mg/dL; and (3) random glucose level > 200 mg/dL. Hypertension was defined as an average systolic blood pressure (SBP) > 140 mmHg or diastolic blood pressure (DBP) > 90 mmHg when measured at least twice, a history of hypertension or use of antihypertensive medication. Cardiovascular disease was defined as the composite of coronary heart disease, angina, congestive heart failure, history of heart attack, and stroke at enrolment. Anaemia among nonpregnant women was defined as < 12 g/dL according to the World Health Organization criteria^22^. Iron deficiency was defined as ferritin < 30 ng/mL^23^. Elevated C-reactive protein (CRP) was considered ≥ 0.3 mg/dL^24^.

### Outcomes

The primary outcome was all-cause mortality obtained from the NHANES data linked to National Death Index data. The participants who were lost to follow-up were censored at their last visit. Survival time was the period from the date of the interview to the date of death or the end of the mortality period. Unnatural death was defined as death caused by accidents, and natural death was defined as death caused by diseases of heart, malignant neoplasms, cerebrovascular diseases, DM, influenza and pneumonia, nephritis, nephrotic syndrome or nephrosis. The secondary outcome was CKD development (a composite of baseline eGFR < 60 ml/min/1.73 m^2^ and/or baseline random urine albumin-to-creatinine ratio (ACR) ≥ 30 mg/g). Specifically, to analyse CKD development, we included subjects who could be identified as having CKD or not having CKD according to the definition of CKD development (*n* = 5334).

### Statistical analysis

All statistical analyses were conducted with SPSS software version 25 (IBM Corp., Armonk, NY, USA) and R software version 4.0.5 (www.r-project.org; R Foundation for Statistical Computing, Vienna, Austria). Categorical variables are described as numbers and percentages. Continuous variables are described as the mean ± standard deviation or as the median with interquartile range (IQR). The Shapiro–Wilk test was used for the normality test. To compare the baseline characteristics according to tertile of log(sTfR), the chi-squared test was performed for categorical variables (Fisher’s exact test if not applicable), while one-way analysis of variance (ANOVA) was performed for continuous variables (Kruskal–Wallis test if not applicable). To calculate *P* for trend, linear-by-linear association was used for categorical variables, and the Jonckheere-Terpstra test was used for continuous variables. The hazard ratio (HR) of all-cause mortality was analysed using multivariable Cox proportional hazards models. Model 1 was a univariable model. In model 2, we adjusted for sociodemographic information (age, race, and education). In model 3, we further adjusted for body mass index (BMI), serum haemoglobin, ferritin, CRP, baseline eGFR, and ACR. In model 4, we further adjusted for risk factors for mortality (DM, hypertension, cardiovascular disease, and cancer). Kaplan–Meier survival curves were drawn, and the log-rank test was used to compare the survival distribution of tertiles of log(sTfR). A cubic spline curve was drawn to show the association between continuous log(sTfR) and all-cause mortality. Multivariable model 4 was applied to the cubic spline curve. The reference point of log(sTfR) was 0.47, the lowest estimated HR in the spline curve. Subgroup analyses were conducted with respect to age, DM, hypertension, CKD (eGFR < 60 ml/min per 1.73 ${\mathrm{m}}^{2}$ or ACR ≥ 30 mg/g), cancer, haemoglobin, ferritin, and CRP. The odds ratio (OR) of CKD development was analysed using multivariable binary logistic regression models. The multivariable models used for primary analysis were used, except eGFR and ACR were excluded. A cubic spline curve was drawn to show the association between continuous log(sTfR) and CKD. *P* < 0.05 was considered statistically significant.

## Results

### Baseline characteristics at study entry

The baseline characteristics are shown in Table 1 according to tertiles of log(sTfR). The median (IQR) patient age was 34.0 years (27.0–42.0). As log(sTfR) increased, the proportion of non-Hispanic black individuals increased, while the proportions of Mexican American, other Hispanic, and non-Hispanic white individuals decreased. BMI, SBP, and DBP increased with log(sTfR). Participants with DM, hypertension, and cardiovascular disease tended to have higher log(sTfR) values. Moreover, participants with cancer tended to have lower log(sTfR) values. The proportion of never smoker increased with log(sTfR). As log(sTfR) increased, haemoglobin and ferritin decreased. ACR, fasting glucose, and CRP tended to increase in the highest tertile of log(sTfR); however, there were few changes between tertiles. Participants in a lower eGFR category tended to have higher log(sTfR) values.

**Table 1:** Baseline participant characteristics.

Variable	log(soluble transferrin receptor)				P for trend
	Total (*n* = 5403)	< 0.45 (*n* = 1,874)	0.45–0.57 (*n* = 1668)	> 0.57 (*n* = 1861)	
Age (year), median (IQR)	34.0 [27.0;42.0]	34.0 [26.0;42.0]	34.0 [26.5;42.0]	34.0 [27.0;42.0]	0.455
**Race/ethnicity, n (%)**					< 0.001
Mexican American	1173 (21.7)	442 (23.6)	353 (21.2)	378 (20.3)	
Other Hispanic	459 (8.5)	170 (9.1)	148 (8.9)	141 (7.6)	
Non-Hispanic White	2386 (44.2)	980 (52.3)	755 (45.3)	651 (35.0)	
Non-Hispanic Black	1098 (20.3)	188 (10.0)	319 (19.1)	591 (31.8)	
Other Race	287 (5.3)	94 (5.0)	93 (5.6)	100 (5.4)	
Body mass index (kg/m^2^)	27.4 [23.4;33.0]	26.0 [22.4;30.9]	27.3 [23.4;32.9]	29.3 [24.7;35.1]	< 0.001
Systolic blood pressure (mmHg)	112.0 [104.0;120.0]	110.0 [104.0;118.0]	112.0 [104.0;120.0]	114.0 [106.0;122.0]	< 0.001
Diastolic blood pressure (mmHg)	68.0 [60.0;76.0]	66.0 [60.0;74.0]	68.0 [60.0;76.0]	70.0 [62.0;78.0]	< 0.001
Diabetes mellitus, *n* (%)	249 (4.6)	62 (3.3)	66 (4.0)	121 (6.5)	< 0.001
Hypertension, n (%)	925 (17.1)	247 (13.2)	276 (16.5)	402 (21.6)	< 0.001
Cardiovascular disease, n (%)	135 (2.5)	39 (2.1)	35 (2.1)	61 (3.3)	0.019
Cancer, *n* (%)	225 (4.2)	90 (4.8)	75 (4.5)	60 (3.2)	0.016
**Education, n (%)**					0.142
High school or lower	2450 (45.3)	847 (45.2)	718 (43.0)	885 (47.6)	
College or graduate	2948 (54.6)	1025 (54.7)	948 (56.8)	975 (52.4)	
**Smoking, n (%)**					< 0.001
Current smoker	1265 (23.4)	541 (28.9)	343 (20.6)	381 (20.5)	
Ex-smoker	732 (13.6)	272 (14.5)	221 (13.3)	239 (12.8)	
Never smoker	3404 (63.0)	1061 (56.6)	1103 (66.2)	1240 (66.7)	
**Laboratory findings, median (IQR)**					
log(soluble transferrin receptor)	0.5 [0.4; 0.6]	0.4 [0.3; 0.4]	0.5 [0.5; 0.5]	0.7 [0.6; 0.8]	< 0.001
Hemoglobin (g/dL)	13.4 [12.5;14.1]	13.5 [12.9;14.1]	13.5 [12.8;14.1]	12.9 [12.0;13.7]	< 0.001
Ferritin (ng/mL)	38.0 [20.0;69.0]	52.0 [32.0;83.0]	40.0 [24.0;70.0]	22.0 [9.0;45.0]	< 0.001
ACR (mg/g)	6.8 [4.6;11.6]	6.5 [4.6;10.5]	6.7 [4.6;11.2]	7.2 [4.7;13.1]	< 0.001
Fasting glucose (mg/dL)	92.0 [86.0;99.0]	92.0 [86.0;99.0]	92.0 [86.0;99.0]	93.0 [87.0;100.0]	0.044
C-reactive protein (mg/dL)	0.3 [0.1; 0.6]	0.2 [0.1; 0.5]	0.3 [0.1; 0.6]	0.3 [0.1; 0.8]	< 0.001
eGFR < 60 ml/min per 1.73 m^2^, *n* (%)	35 (0.7)	5 (0.3)	10 (0.6)	20 (1.1)	0.002

*ACR* random urine albumin-to-creatinine ratio, *eGFR* estimated glomerular filtration rate.

### Soluble transferrin receptor and mortality

**Figure 2:**
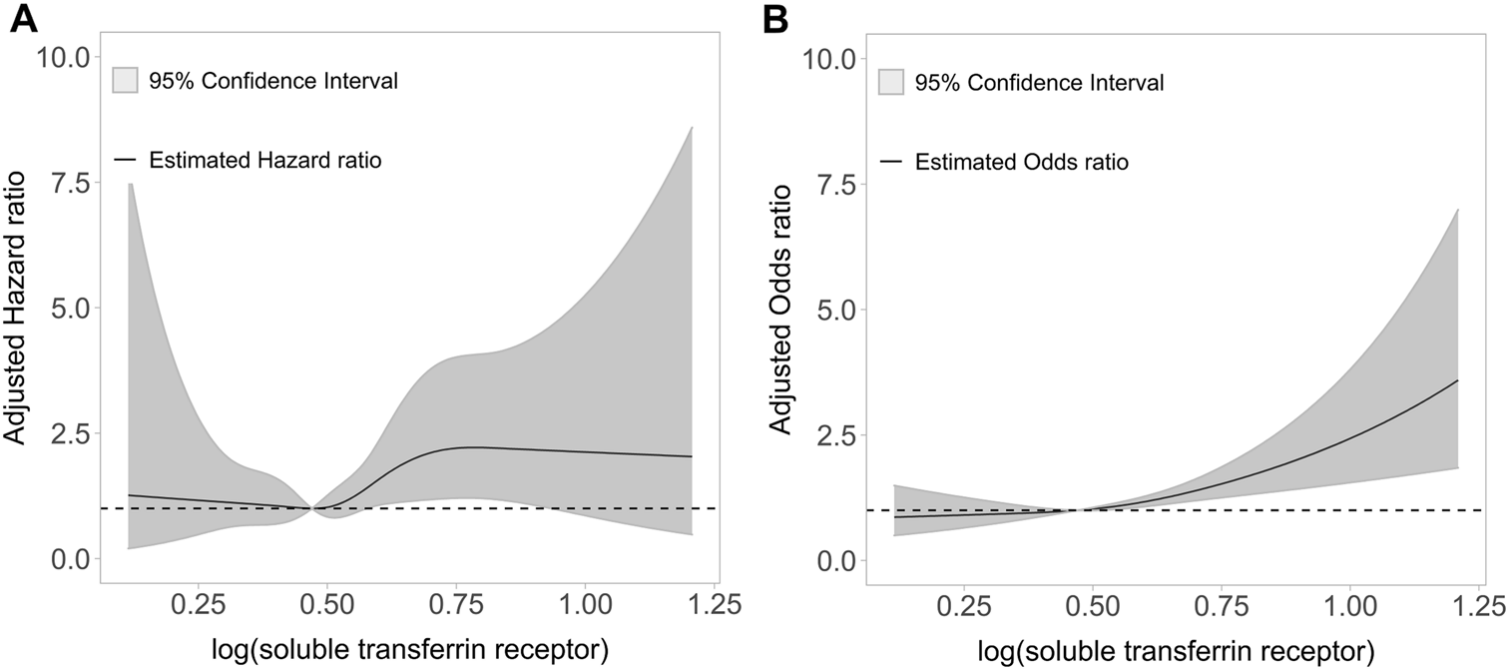
The association of log(soluble transferrin receptor) with all-cause mortality (**A**) and chronic kidney disease development (**B**). (**A**) A cubic spline curve showed that a high soluble transferrin receptor (sTfR) was associated with a high risk of all-cause mortality. A curve represents multivariable hazard ratio. Hazard ratios were adjusted for age, race, education, body mass index (BMI), haemoglobin, ferritin, C-reactive protein (CRP), baseline estimated glomerular filtration rate, random urine albumin-to-creatinine ratio, diabetes mellitus (DM), hypertension, cardiovascular disease, and cancer. (**B**) A cubic spline curve demonstrated an association between high sTfR and CKD development in a multivariable binary logistic regression model. Odds ratio was adjusted for age, race, education, BMI, haemoglobin, ferritin, CRP, DM, hypertension, cardiovascular disease, and cancer.

During a median (IQR) follow-up of 8.7 years (6.7 – 10.8 years), 103 (1.9%) participants died. In the Kaplan–Meier curve, survival in the highest tertile of log(sTfR) was shown to be the lowest (Fig. 1). The incidence rates of all-cause death from the lowest to the highest tertiles of log(sTfR) were 1.9, 1.5, and 3.0 per 1,000 person-years, respectively. The adjusted HRs (95% confidence intervals) of all-cause death (compared with that of the second tertile) were 1.09 (0.62–1.93), and 1.77 (1.05–2.98) for the first and third tertiles of log(sTfR), respectively (Model 4 in Table 2). The spline curve showed that high sTfR was associated with a high risk of all-cause mortality (Fig. 2A). The leading cause of death was malignant neoplasms (32.0%), followed by diseases of the heart (10.7%) and accidents (7.8%) (Supplemental Table 1). The proportion of deaths related to cancer or infection was highest among patients in the highest tertile of sTfR (Supplemental Table 2).

**Table 2:** Risk of all-cause mortality according to tertile of log(soluble transferrin receptor).

	log (soluble transferrin receptor)					
	T1 (< 0.45)		T2 (0.45–0.57)		T3 (> 0.57)	
	HR (95% CI)	*P*	HR (95% CI)	*P*	HR (95% CI)	*P*
Model 1	1.31 (0.76–2.27)	0.328	1.0 (reference)		1.98 (1.21–3.24)	0.006
Model 2	1.25 (0.72–2.17)	0.419	1.0 (reference)		1.87 (1.14–3.07)	0.013
Model 3	1.09 (0.61–1.92)	0.764	1.0 (reference)		1.78 (1.05–3.00)	0.030
Model 4	1.09 (0.62–1.93)	0.478	1.0 (reference)		1.77 (1.05–2.98)	0.032
Number of events (%)	29 (1.5)		23 (1.4)		51 (2.7)	

*HR* hazard ratio, *CI* confidence interval.

Model 1: unadjusted.

Model 2: Adjusted for age, race, and education.

Model 3: Model 2 + body mass index, serum hemoglobin, ferritin, C-reactive protein, baseline estimated glomerular filtration rate, and random urine albumin-to-creatinine ratio.

Model 4: Model 3 + diabetes mellitus, hypertension, cardiovascular disease, and cancer.

### Soluble transferrin receptor and chronic kidney disease

CKD developed in 120 (6.5%), 131 (8.0%), and 190 (10.3%) patients in the lowest to the highest tertiles of log(sTfR), respectively (Table 3). The adjusted ORs of CKD (compared to that of the second tertile) were 0.82 (0.63–1.06) and 1.28 (1.00–1.65) for the first and third tertiles of log(sTfR), respectively (Model 3 in Table 3). The cubic spline curve demonstrated an association between high sTfR and CKD development (Fig. 2B).

**Table 3:** Risk of a chronic kidney disease development according to tertile of log(soluble transferrin receptor).

	log (soluble transferrin receptor)					
	T1 (< 0.45)		T2 (0.45–0.57)		T3 (> 0.57)	
	OR (95% CI)	*P*	OR (95% CI)	*P*	OR (95% CI)	*P*
Model 1	0.79 (0.61–1.03)	0.086	1.0 (reference)		1.33 (1.05–1.67)	0.017
Model 2	0.80 (0.62–1.04)	0.100	1.0 (reference)		1.30 (1.02–1.64)	0.029
Model 3	0.82 (0.63–1.06)	0.143	1.0 (reference)		1.28 (1.00–1.65)	0.045
Model 4	0.83 (0.63–1.08)	0.175	1.0 (reference)		1.22 (0.95–1.57)	0.118
Number of events (%)	120 (6.5)		131 (8.0)		190 (10.3)	

*OR* odds ratio, *CI* confidence interval.

Model 1: unadjusted.

Model 2: Adjusted for age, race, and education.

Model 3: Model 2 + body mass index, serum hemoglobin, ferritin, and C-reactive protein.

Model 4: Model 3 + diabetes mellitus, hypertension, cardiovascular disease, and cancer.

### Subgroup analyses

**Figure 3:**
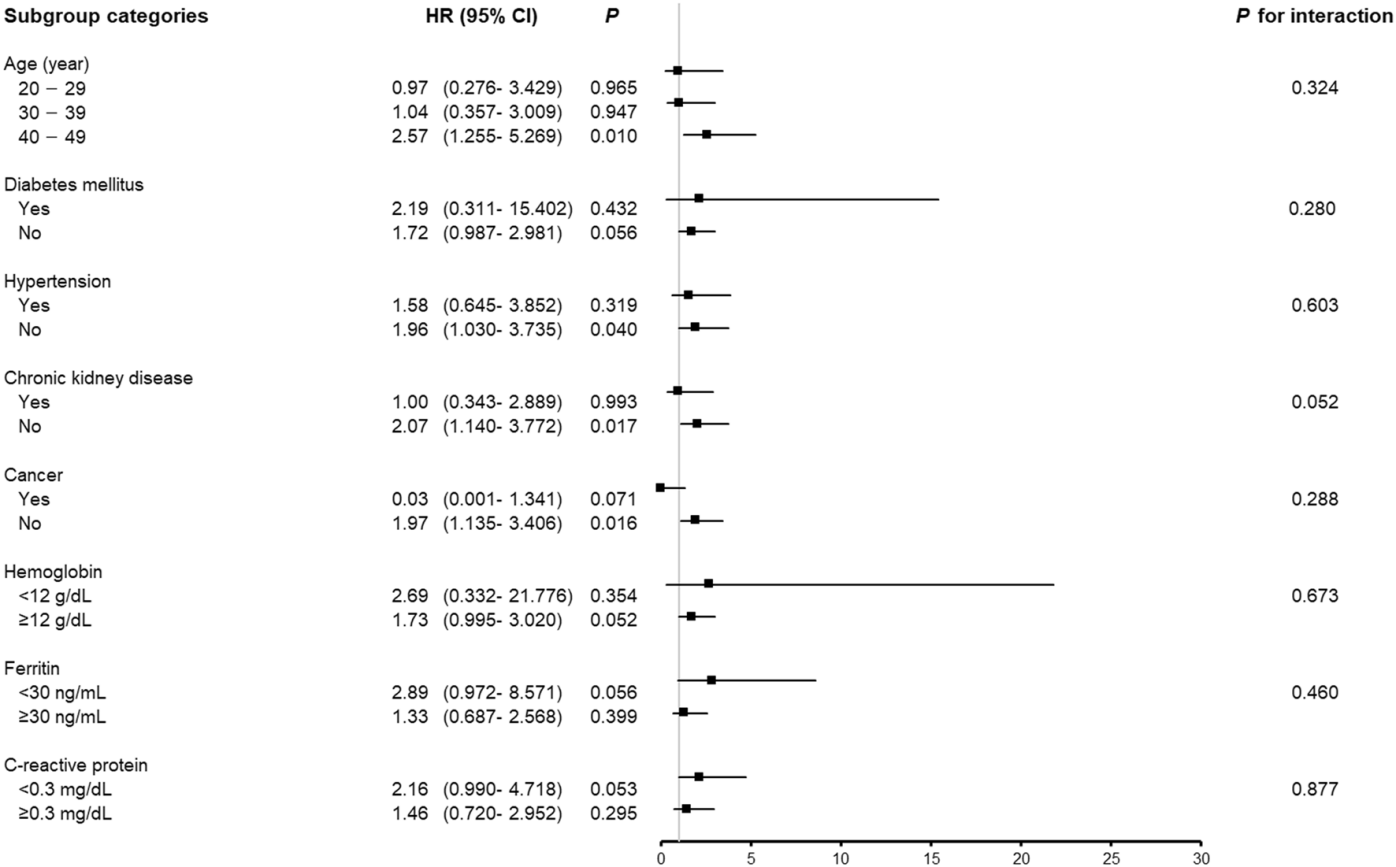
Subgroup association of soluble transferrin receptor with all-cause mortality. The risks for all-cause mortality of the highest tertile (log(sTfR) > 0.57) and the second tertile (log(TfR) 0.45–0.57) are shown. Hazard ratios (HRs) were adjusted for age, race, education, body mass index, haemoglobin, ferritin, C-reactive protein, baseline estimated glomerular filtration rate, random urine albumin-to-creatinine ratio, diabetes mellitus, hypertension, cardiovascular disease, and cancer. *HR* hazard ratio, *CI* confidence interval.

The risk of individuals in the highest tertile of log(sTfR) was compared with the risk of individuals in the second tertile of log(sTfR) by subgroups stratified according to age (20–29, 30–39, or 40–49 years old), DM (yes or no), hypertension (yes or no), CKD (yes or no), cancer (yes or no), haemoglobin (< 12 or ≥ 12 g/dL), ferritin (< 30 or ≥ 30 ng/mL), and CRP (< 0.3 or ≥ 0.3 mg/dL). *P* values for interactions were > 0.05 for all of the subgroups, suggesting that the increased risk of all-cause mortality associated with sTfR was clear regardless of age, DM, hypertension, CKD, cancer, anaemia, iron deficiency, and CRP (Fig. 3). High sTfR was associated with a higher risk of all-cause mortality particularly in participants in their 40 s, in those without hypertension, in those without CKD, and in those without cancer.

### Sensitivity analyses

For sensitivity analyses, we drew cubic spline curves while changing the reference. When the reference points of log(sTfR) were 0.40, 0.45, and 0.50, cubic spline curves showed that high sTfR was associated with all-cause mortality (Supplemental Fig. 2). In addition, to assess whether the association between sTfR and mortality was driven by an association of sTfR with natural death rather than unnatural death, we compared the HRs of sTfR for natural vs unnatural death. The HR for natural death among patients with the highest tertile of log(sTfR) was 2.70 in model 4 (Supplemental Table 3). In contrast, as shown in Supplemental Table 4, unnatural mortality was not associated with high sTfR.

## Discussion

 Although there has been much interest in the clinical significance of sTfR, studies on the mortality of sTfR in the general population are rare. Our long-term analysis of a general population of US adult females revealed that high sTfR was associated with a significantly increased risk of all-cause mortality regardless of anaemia and iron storage status. We also showed that sTfR was significantly associated with CKD. The significantly increased risk of all-cause mortality associated with high sTfR was particularly pronounced in subgroups of participants in their 40 s, in those without hypertension, in those without CKD, and in those without cancer, suggesting that high sTfR might warrant monitoring in people without underlying disease; however, clinical studies are needed.

Previous studies have shown the association between high sTfR and mortality mainly in high-risk patients, such as those with heart failure^11–13^, diabetic patients with coronary artery disease^14^, and patients with coronary heart disease^15^. Moreover, studies of sTfR and mortality in the general population are rare. One study of 1864 individuals from the general German population showed that high sTfR was associated with all-cause mortality and cardiovascular mortality^16^. However, this study was restricted to hospital-based participants scheduled for coronary angiography. In addition, the study was limited to participants of German ancestry, and it did not include a variety of ethnic groups. Due to these factors, it is difficult to conclude that the results are representative of the general population. Similar to our results, in this previous study, the relationship between sTfR and mortality was nonlinear and had a J shape in the spline curves. However, the association between low sTfR and mortality was not statistically significant. In the multivariable model, anaemia was included as a covariate; however, ferritin, which is indicative of iron storage^25^, was not included in the previous study. In contrast, in our study, the association between high sTfR and all-cause mortality was evident regardless of anaemia and iron storage status. Even if haemoglobin and ferritin were adjusted, the association between high sTfR and all-cause mortality was evident. In addition, in the subgroup analysis, *P* for interaction was nonsignificant for the subgroups stratified according to ferritin and haemoglobin. These results suggest that sTfR is an independent factor related to mortality in this study, independently of anaemia and iron storage.

In this study, the risk of all-cause mortality in the highest tertile (log(sTfR) > 0.57) was 1.7 times higher than that in the reference group. The first possible mechanism to explain this is that sTfR is associated with a hypoxic environment^8,26,27^, which is a risk factor for mortality^28,29^. In hypoxic cells, the expression of sTfR increased as mediated by hypoxia-inducible factors^26,27^. In addition, sTfR was elevated in chronic hypoxic diseases such as cystic fibrosis in a clinical study^8^. Second, there is a relationship between TfR and chronic diseases such as DM^9^, CKD^10^, heart failure^11^, and cancer^30,31^. In our study, high sTfR was associated with CKD. These pathological states contribute to mortality. However, our study showed that even in patients without CKD, hypertension, or cancer, high sTfR was associated with mortality. This result suggests that there are other mechanisms by which high sTfR can contribute to mortality apart from comorbidities. For example, TfR might have played an important role in adaptive immunity^32,33^. According to whole-genome sequencing, if people have a homozygous p.Tyr20His substitution in TfR, the internalization of TfR into the cell is hindered, and the expression of TfR on the cell surface is increased^32^. This was expressed as an immunological defect because TfR-mediated iron uptake was important for lymphocyte proliferation and development^33^. Therefore, in the case of people whose TfR expression increased due to such a genetic mutation, there might be a problem with adaptive immunity and thus mortality might be high. In addition, because TfR could be a mediator of virus penetration into cells^34^, people with increased expression of TfR might be susceptible to viral infection. In our study, there was an excess of deaths related to infection or cancer among patients in the high sTfR tertile.

The HR of all-cause mortality in the group with the highest of sTfR was less than 2 in our study. The reason for the low HR is that we analysed the general population rather than high-risk patients. In a previous study on the general population in Germany^16^, the HR for all-cause mortality in the group with the highest sTfR was 1.76, which is similar to the corresponding HR of the present study.

The strength of this study includes the inclusion of a variety of races and the fact that the population was not a hospital-based population; therefore, there was relatively little selection bias. To our knowledge, there was only one study on individuals of German ancestry that analysed high sTfR and mortality in the general population^16^; therefore, this is the first study that can represent a more diverse general population in the analysis of the association between high sTfR and mortality. In addition, this study is a larger-scale study than previous studies on this subject. Nevertheless, this study also has several limitations. This was an observational study. Therefore, we could not control for all confounders. However, to control for confounders, we adjusted for several variables that could affect the relationship between sTfR and mortality. We found that the relationship between sTfR and mortality was significant. In addition, when subgroup analysis was performed, the *P* values for the interactions of confounders were all nonsignificant. This finding confirmed that these confounders did not act as modified effectors on the relationship between sTfR and all-cause mortality. Because sTfR was measured only in women in NHANES, the association between sTfR and mortality in men was not analysed. Nevertheless, the clinical validity of the sTfR test cannot be overlooked, as a previous study involving the general population showed that high sTfR was associated with increased mortality in older men and women^16^. More studies are needed to support the clinical validity of the sTfR test. Due to the classification of cause of death in the NHANES dataset, we could not investigate in detail unnatural death and death related to infection.

In conclusion, high sTfR is associated with all-cause mortality in the general population regardless of anaemia and iron storage status. In particular, risk was most pronounced in participants in their 40 s, in those without hypertension, in those without CKD, and in those without cancer. Our findings suggest that sTfR is a predictor of mortality.

## Supplementary Information

Supplementary Information.
