# Supplementary material for: Soluble transferrin receptor can predict all-cause mortality regardless of anaemia and iron storage status
Source: Sci Rep. 2022 Jul 13;12:11911. doi: 10.1038/s41598-022-15674-w (PMC9279452; doi:10.1038/s41598-022-15674-w)
Supplement: Supplementary file 1 — Supplementary Information. [file 41598_2022_15674_MOESM1_ESM.docx]

**Soluble transferrin receptor can predict all-cause mortality regardless of anemia and iron storage: Results from the National Health and Nutrition Examination Survey, 2003 to 2010**

**Supplemental Figure 1 to 2**

**Supplemental Table 1 to 4**

**Corresponding author:**

Jung Pyo Lee (jungpyolee@snu.ac.kr)

**Contents**

**Supplemental Figure 1.** …………………………………………………………………………2

**Supplemental Figure 2.** …………………………………………………………………………3

**Supplemental Table 1.** …………………………………………………………………………4

**Supplemental Table 2.** …………………………………………………………………………5

**Supplemental Table 3.** …………………………………………………………………………6

**Supplemental Table 4.** …………………………………………………………………………7

**Supplemental Figure 1. Flow diagram of study cohort.**


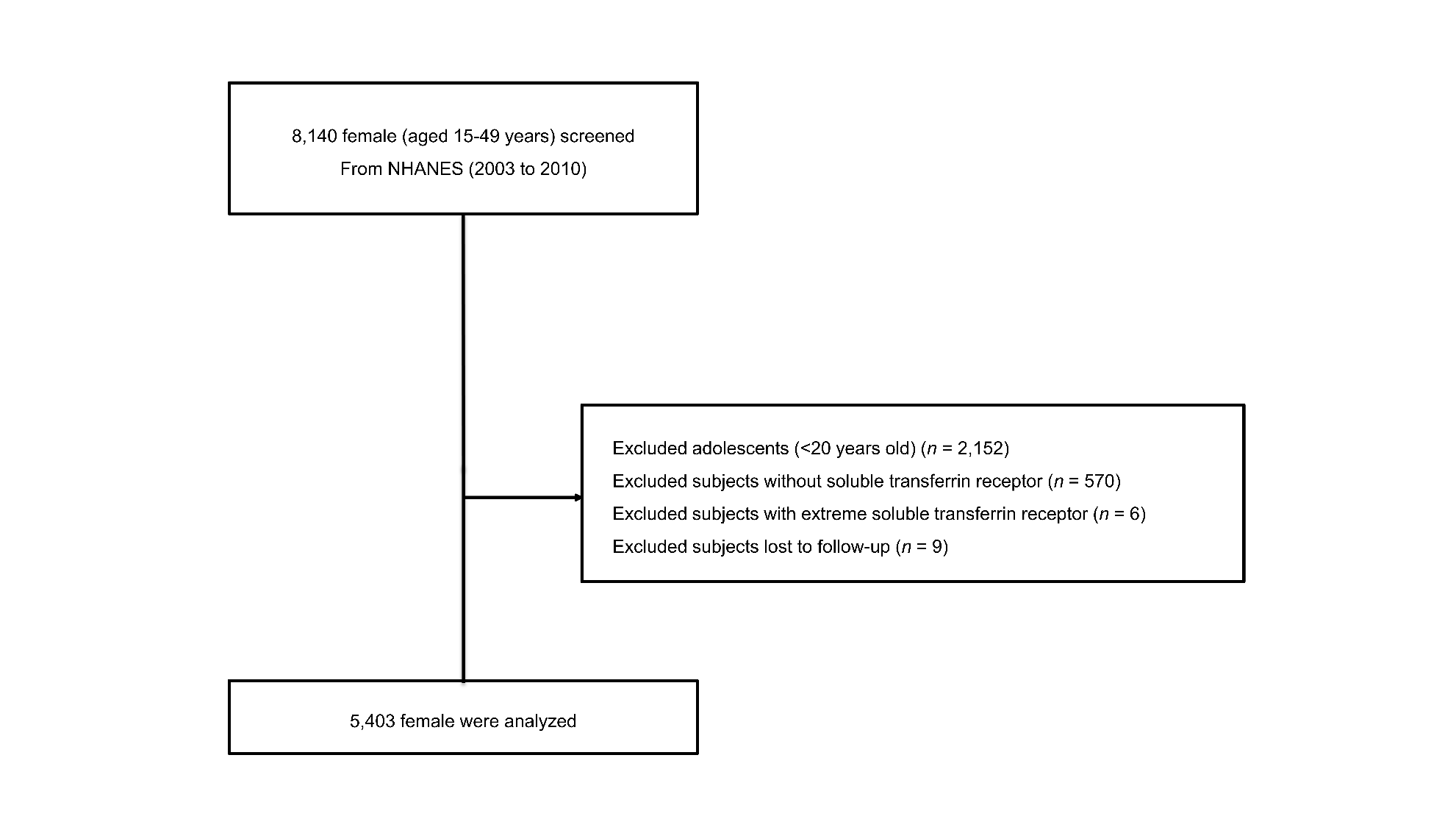


**Supplemental Figure 2. The relationship between the soluble transferrin receptor and all-cause mortality.** Cubic spline curves showed that high soluble transferrin receptor (sTfR) was associated with a high risk of all-cause mortality when the reference points of log (sTfR) were 0.40 (A), 0.45 (B), and 0.50 (C). Curves represent multivariable hazard ratios. Hazard ratios were adjusted for age, race, education, body mass index, hemoglobin, ferritin, C-reactive protein, baseline estimated glomerular filtration rate, random urine albumin-to-creatinine ratio, diabetes mellitus, hypertension, cardiovascular disease, and cancer.


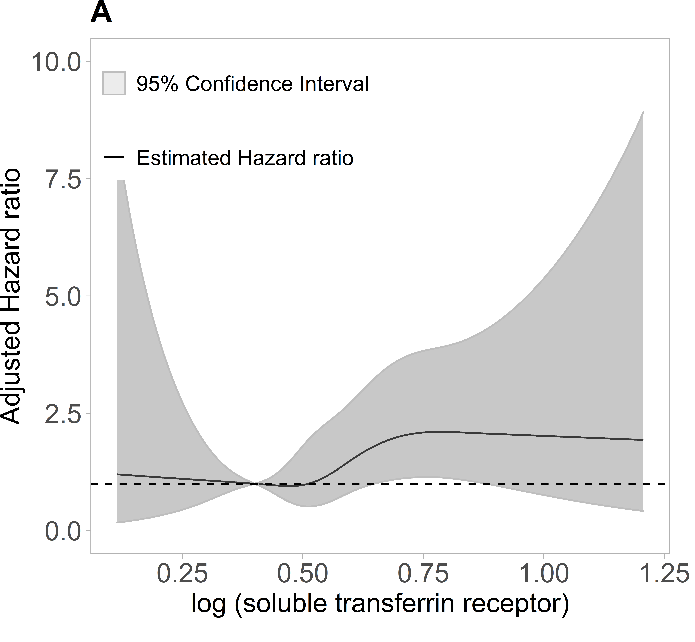

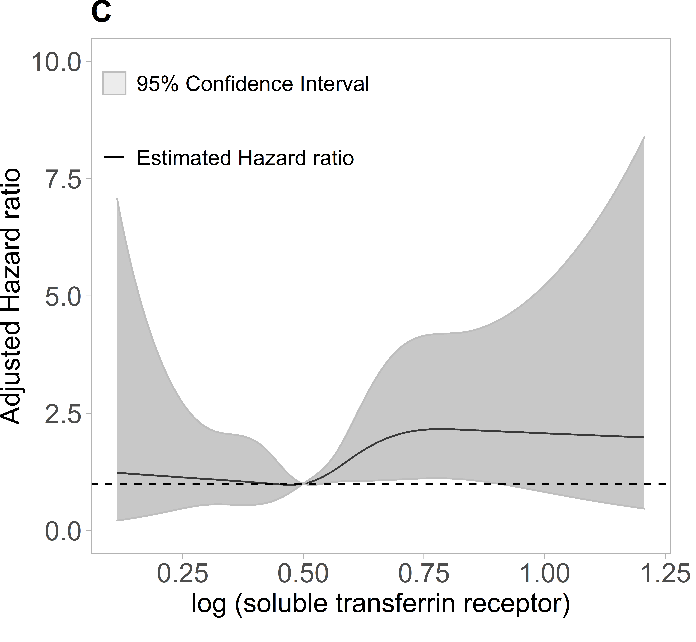

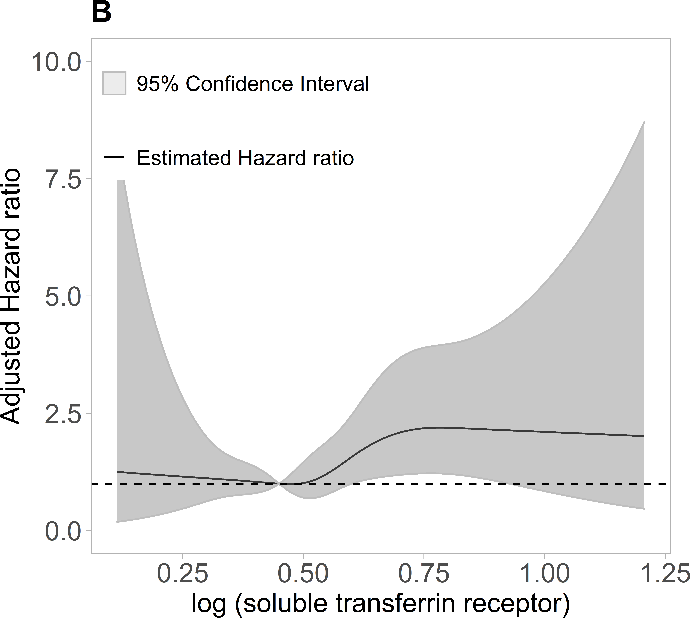


**Supplemental Table 1. Proportions of causes of death**

| Causes of death | n (%) |
| --- | --- |
| Diseases of heart | 11 (10.7) |
| Malignant neoplasms | 33 (32.0) |
| Accidents (unintentional injuries) | 8 (7.8) |
| Cerebrovascular diseases | 3 (2.9) |
| Diabetes mellitus | 2 (1.9) |
| Influenza and pneumonia | 2 (1.9) |
| Nephritis, nephrotic syndrome and nephrosis | 1 (1.0) |
| All other causes (residual) | 43 (41.7) |

% represents the ratio of specific causes to total death.

**Supplemental Table 2. Proportions of deaths related to cancer or infection according to log(soluble transferrin receptor)**

|  | log(soluble transferrin receptor) | | | | |
| --- | --- | --- | --- | --- | --- |
| Variable | | Total deaths (n =103) | <0.45 (n = 29) | 0.45–0.57 (n = 23) | >0.57 (n = 51) |
| Deaths related to cancer or infection | | 35 (33.9) | 8 (27.5) | 5 (21.7) | 22 (43.1) |

Death due to infection was defined as death caused by influenza and pneumonia.

**Supplemental Table 3. Risk of natural mortality according to the tertile of log(soluble transferrin receptor)**

|  | log(soluble transferrin receptor) | | | | | |
| --- | --- | --- | --- | --- | --- | --- |
|  | T1 (<0.45) | | T2 (0.45–0.57) | | T3 (>0.57) | |
|  | HR (95% CI) | *P* | HR (95% CI) | *P* | HR (95% CI) | *P* |
| Model 1 | 1.37 (0.60–3.14) | 0.451 | 1.0 (reference) |  | 2.59 (1.26–5.33) | 0.009 |
| Model 2 | 1.34 (0.58–3.08) | 0.484 | 1.0 (reference) |  | 2.39 (1.16–4.93) | 0.018 |
| Model 3 | 1.11 (0.47–2.63) | 0.808 | 1.0 (reference) |  | 2.69 (1.27–5.70) | 0.010 |
| Model 4 | 1.12 (0.47–2.65) | 0.796 | 1.0 (reference) |  | 2.70 (1.27–5.71) | 0.009 |

HR, hazard ratio; CI, confidence interval

Model 1: Unadjusted

Model 2: Adjusted for age, race, and education

Model 3: Model 2 + body mass index, serum haemoglobin, ferritin, C-reactive protein, baseline estimated glomerular filtration rate, and random urine albumin-to-creatinine ratio

Model 4: Model 3 + diabetes mellitus, hypertension, cardiovascular disease, and cancer

**Supplemental Table 4. Risk of unnatural mortality according to the tertile of log(soluble transferrin receptor)**

|  | log(soluble transferrin receptor) | | | | | |
| --- | --- | --- | --- | --- | --- | --- |
|  | T1 (<0.45) | | T2 (0.45–0.57) | | T3 (>0.57) | |
|  | HR (95% CI) | *P* | HR (95% CI) | *P* | HR (95% CI) | *P* |
| Model 1 | 0.29 (0.03–2.64) | 0.275 | 1.0 (reference) |  | 0.67 (0.15–3.01) | 0.275 |
| Model 2 | 0.24 (0.02–2.24) | 0.214 | 1.0 (reference) |  | 0.76 (0.17–3.41) | 0.723 |
| Model 3 | 0.24 (0.02–2.28) | 0.218 | 1.0 (reference) |  | 0.75 (0.15–3.66) | 0.731 |
| Model 4 | 0.24 (0.02–2.30) | 0.220 | 1.0 (reference) |  | 0.77 (0.16–3.69) | 0.745 |

HR, hazard ratio; CI, confidence interval

Model 1: Unadjusted

Model 2: Adjusted for age, race, and education

Model 3: Model 2 + body mass index, serum haemoglobin, ferritin, C-reactive protein, baseline estimated glomerular filtration rate, and random urine albumin-to-creatinine ratio

Model 4: Model 3 + diabetes mellitus, hypertension, cardiovascular disease, and cancer
